# Effects of Polypropylene Fibre and Strain Rate on Dynamic Compressive Behaviour of Concrete

**DOI:** 10.3390/ma12111797

**Published:** 2019-06-03

**Authors:** Meng Chen, Chenhui Ren, Yangbo Liu, Yubo Yang, Erlei Wang, Xiaolong Liang

**Affiliations:** 1 School of Resource and Civil Engineering, Northeastern University, Shenyang 110819, China; chenmeng@mail.neu.edu.cn (M.C.); renchenhui97@163.com (C.R.); 20161924@stu.neu.edu.cn (Y.L.); 20161993@stu.neu.edu.cn (Y.Y.); liangliforever@aliyun.com (X.L.); 2 State Key Laboratory of Silicate Materials for Architectures, Wuhan University of Technology, Wuhan 430070, China; 3 Design & Research Institute of Wuhan University of Technology, Wuhan 430070, China

**Keywords:** Fibre reinforced concrete, polypropylene, impact loading, constitutive model, failure mode

## Abstract

This paper presents an experimental study on the dynamic compressive behaviour of polypropylene (PP) fibre reinforced concrete under various strain rates using split Hopkinson pressure bar (SHPB) equipment. The effects of PP fibre content and strain rate on the dynamic compressive stress-strain relationship and failure patterns were estimated. The results indicated that the addition of PP fibre enhanced the dynamic compressive properties of concrete mixtures although it resulted in a significant reduction in workability and a slight decrease in static compressive strength. Considering the workability, static compressive strength and dynamic compressive behaviour, the optimal PP fibre content was found to be 0.9 kg/m^3^ as the mixture exhibited the highest increase in dynamic compressive strength of 5.6%, 40.3% in fracture energy absorption and 11.1% in total energy absorption; further, it showed the least reduction (only 5.8%) in static compressive strength among all mixtures compared to the reference mixture without fibre. For all mixtures, the dynamic compressive properties, energy absorption capacity, strain at peak stress, ultimate strain and dynamic increase factor (DIF) were significantly influenced by strain rate, i.e., strain rate effect. When the strain rate was relatively low, PP fibres were effective in controlling the cracking, and the dynamic compressive properties of PP fibre reinforced mixtures were improved accordingly.

## 1. Introduction

Concrete is the most widely used construction material in the world [1]. However, normal concrete is inherently brittle when subjected to static (including tensile and flexural) and high-velocity dynamic loadings, and is susceptible to cracking induced by unsuitable curing conditions, freeze-thawing and shrinkage [2,3,4,5]. As a result, normal concrete exhibits a brittle failure pattern under dynamic impact loadings, posing a serious threat to the integrity and safety of concrete infrastructure in highly seismic (e.g., earthquake) or marine zones (e.g., wave impact and wind load). Moreover, high-strength concrete is usually supplied by the protective structures under dynamic impact loading. In addition, many cracks may appear on the concrete surfaces under impact loadings and thus provide transport pathways for some aggressive ions (e.g., chloride ions), which would facilitate the corrosion of reinforcing steel and thus adversely affect the durability of reinforced concrete structures.

In order to overcome the aforementioned shortcomings, many researchers have attempted to incorporate randomly distributed short fibres (i.e., steel, polypropylene and basalt fibres) into the concrete matrix. The inclusion of these short fibres into the concrete results in a significant increase of ductility, impact resistance and energy absorption capacity [6,7,8,9]. The enhancement of these material properties can be ascribed to the uniform distribution of short fibres in the concrete matrix [10,11]. Generally, these materials are classified as fibre reinforced concrete (FRC) [12]. Among all of these fibres, steel fibres were first introduced as the reinforcement in concrete [13] and showed high efficiency in improving the dynamic properties of concrete [9,12,14,15]. Karimi et al. [16] explored the fracture energy of three types asphalt concrete and concluded that the mixtures with a 0.2% and 1.5% SWF (steel wool fibre) showed lower fracture energy than the neat mixture. Wu et al. [9] investigated the dynamic behaviour of hybrid steel fibre reinforced ultra-high-performance concrete (UHPC) and concluded that UHPC with hybrid long (1.5%) and short (0.5%) steel fibre reinforcement exhibited the best dynamic mechanical properties. Hao et al. [12] explored the dynamic compressive behaviour of concrete reinforced with randomly distributed spiral steel fibres and found that the energy absorption capacity of the tested concrete was significantly increased with increasing steel fibre dosage. However, some drawbacks of steel fibres may limit the large-scale engineering application of steel fibre reinforced concrete around coastal zones. For instance, as steel fibres rust easily and its weight may cause problems such as fibre balling during the mixing process [17]. When the coastal concrete structures suffer from impact such as wave impact and wind load, the drawbacks of steel fibres facilitate the corrosion of RC structures. Therefore, it is vital to incorporate other types of fibres such as synthetic fibres into normal concrete to improve its dynamic compressive properties as well as corrosion resistance, thereby improving the durability of RC structures near marine zones made by this material.

In the past decades, polypropylene (PP) fibre has been increasingly used in concrete owing to its advantages of lightweight and high corrosion resistance [18]. It was observed that PP fibre was effective in reducing the plastic shrinkage and improving the impact resistance of the matrix [3,18,19]. In recent years, several studies have been conducted on the dynamic properties of PP fibre reinforced concrete. Zhang et al. [20] investigated the dynamic behaviour of PP fibre reinforced mortar under compressive impact loading using SHPB. It was found that the dynamic behaviour of PP fibre reinforced mortar was significantly influenced by strain rate. Moreover, PP fibres can improve the impact toughness of the matrix. Jahanbakhsh and Karimi et al. [21,22,23]. considered the fracture energy as a material property that is independent of sample size and geometry. Moreover, they calculated the fracture energy according to RILEM TC50-FMC specifications by dividing the fracture work. Zhang et al. [24] conducted drop weight test to estimate the flexural impact of PP fibre reinforced concrete and suggested that adding PP fibres into the concrete can enhance its impact resistance though a brittle failure pattern was observed. Fu et al. [2] also found that the addition of PP fibres improved the energy absorption capacity of the concrete matrix. However, different volume fractions of PP fibre should be considered in order to determine the optimal PP fibre content in normal concrete matrix under the dynamic loading with high strain rates. Although Zhang et al. [17] concluded that the optimal content of PP fibre was 1.5 kg/m^3^, which was drawn mainly based on the experimental results of static mechanical tests. In addition, it is stated that the optimal fibre volume fraction for the macro-fibre is 0.5% [25]. Nevertheless, the optimal content for PP fibre is still not determined. Moreover, a number of studies examining the dynamic properties of PP fibre reinforced concrete utilised SHPB equipment with a high strain rate (i.e., normally in the range between 10^2^ s^−1^ and 10^3^ s^−1^ [12,26,27,28]), as the previous study mainly focused on the drop weight test. However, the method of drop weight test is greatly restricted by the specimen size and configuration [9]. In conclusion, the dynamic compressive properties of PP fibre reinforced concrete mixtures with different PP fibre volume fractions under high strain rate have not been extensively studied. Therefore, it is important to investigate the dynamic compressive behaviour of concrete reinforced with different dosages of PP fibres.

The main purpose of this paper is to provide a better understanding of the effects of PP fibre dosage and strain rate on the dynamic compressive behaviour of concrete. Portland cement was used as the main binder material and the compressive strength of all mixture was around 60 MPa. PP fibres with various content (i.e., 0.9, 1.8, 2.7 and 3.6 kg/m^3^) were used. The PP fibre content of 0.9 kg/m^3^ approximately equals to 0.1% fibre volume fraction (*V_f_*). Thus, fibre content of 1.8, 2.7, 3.6 kg/m^3^ roughly corresponds to 0.2, 0.3, 0.4% *V_f_*. Firstly, the slump test was conducted to determine the workability of fresh mixture and the static compressive strength was evaluated. Subsequently, the dynamic compressive properties including stress-strain curve and failure mode were examined using SHPB. The dynamic increase factor (DIF), fracture energy, total energy, the strain at peak stress and ultimate strain were discussed in detail by comparing the difference for all mixtures. The fitted DIF functions for each concrete mixture were derived based on statistical study for providing guidance on incorporating PP fibres to the cement matrix. Finally, PP fibre reinforced mixtures in the form of scanning electron microscopy (SEM) images were presented to further understand the enhancement of dynamic compressive behaviour after incorporating fibres.

## 2. Experimental Program

### 2.1. Raw Materials

**Figure 1:**
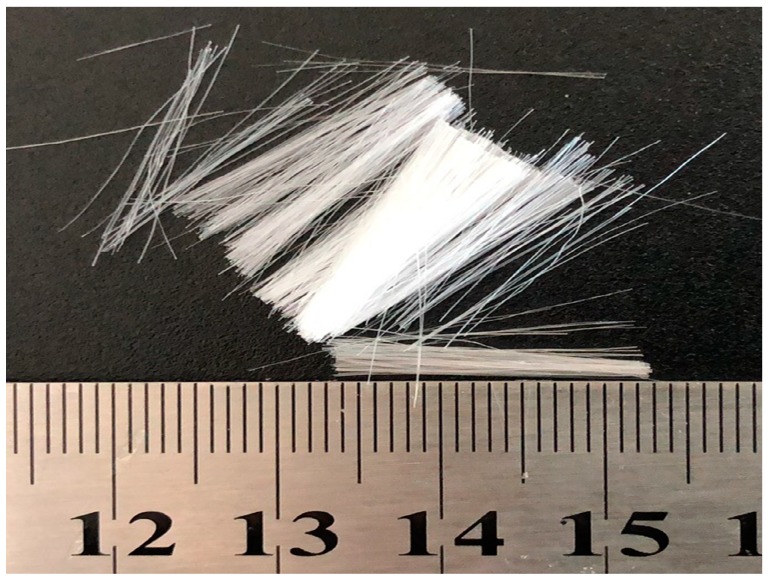
Photograph of PP fibre.

Portland cement (P.I. 42.5), bought from Liaoning Shan Shui Gong Yuan, Co., Ltd. (Liaoning, China), was used as the main binder material with chemical compositions listed in in Table 1. The specific gravity of cement is 3.09. Natural river sand with a nominal maximum size of 4.75 mm was used as the fine aggregate, the specific gravity of which is 2.56. Coarse aggregates were prepared by mixing two different maximum particle sizes of crushed granite, which were 5 mm and 20 mm, respectively. Fine and coarse aggregates were used in saturated surface dry (SSD) conditions according to ASTM C128-15 [29] and ASTM C127-15 [30]. The particle size distributions of fine and coarse aggregates are presented in Table 2 and Table 3, respectively. Multifilament polypropylene (PP) fibre (see Figure 1), bought from Beijing Kenye Trade Co., Ltd. (Beijing, China), was used and its properties are shown in Table 4. Polycarboxylate-based superplasticisers (SPs) were used to improve the workability of the mixture.

### 2.2. Mixture Proportions

The water-to-binder ratio (w/b) was adjusted and selected 0.24 for the plain concrete matrix in order to ensure its compressive strength higher than 60 MPa. Based on the previous study [25], the highest fibre content was adopted as 3.6 kg/m^3^ (equivalent to 0.4% fibre volume fraction). The mixture proportions of concrete used in this study are given in Table 5 and marked with specific symbols. The label ‘PP’ represents polypropylene fibre, while ‘00’, ‘09’, ‘18’, ‘27’ and ‘36’ stand for the dosage of the corresponding fibre. For example, the fibre content for the mixture No.1 is 0.0 kg/m^3^, while the fibre content for the mixture No. 2 is 0.9 kg/m^3^.

### 2.3. Specimen Preparation

The mixing procedure is conducted using a SJD30 concrete horizontal-axle mixer. Cement, fine aggregate and coarse aggregate were first dry mixed for 2 min. Water and SPs were then added to the mixture after 2 min dry mix. Since multifilament fibres are difficult to be dispersed uniformly in the mixture [31], PP fibres were gradually added to the wet mix in order to ensure uniform dispersion of fibres. All the fresh mixtures were then cast into moulds for static and dynamic compressive tests. All specimens were demoulded after 24 h and then stored in a standard curing room (20 ± 2 °C and 95% RH) for 28 days. Table 6 shows the experimental program including the number of static specimens, dynamic specimens and strain rate

### 2.4. Test Methods

#### 2.4.1. Workability and Static Compressive Test

The slump test was conducted in accordance with ASTM C143-15a [32] to determine the workability of all fresh concrete mixture, where the test involved in measuring the vertical displacement difference between the top of the testing mould and top surface of the fresh concrete specimen. Three 150 mm side-length cubic specimens of each mixture were used for static compression test using a universal testing machine (Zhejiang Luda Machinery Instrument Co., Ltd., Zhejiang, China) at the testing age of 28 d according to Chinese standard GB/T50081-2002 [33].

#### 2.4.2. Dynamic Compressive Test

**Figure 2:**
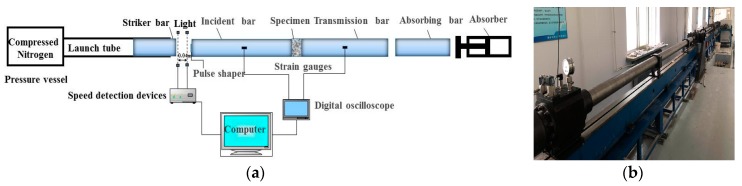
Schematic diagram and image of SHPB testing system: (**a**) Schematic diagram of SHPB testing system; (**b**) Image of SHPB testing apparatus.

In this study, the dynamic compressive properties of all concrete mixtures were evaluated by 100 mm diameter SHPB testing equipment at Northeastern University, Liaoning, China. The SHPB testing system is shown in Figure 2 The striker, incident, transmission and absorbing bars are all made of 100 mm diameter superior alloy steel. The lengths of striker, incident, transmission and absorbing bars are 600, 5000, 3500 and 1200 mm, respectively. Cylindrical specimens 100 mm in diameter by 50 mm in height were used for each mixture in this study.

Regarding the testing procedure, the specimen was first placed between the incident and transmission bars, as shown in Figure 2a when the ‘start’ button in the computer system was pressed, the 100 mm diameter striker bar impacted the incident bar under the set compressing nitrogen to generate the incident pulse ($\varepsilon_{i}\left(t\right)$), known as incident wave. Afterwards, the specimen started to deform when the incident pulse reached it. At that time, some of the incident pulse was reflected as the reflected pulse ($\varepsilon_{r}\left(t\right)$). The remaining pulse passed through the specimen and propagated to the transmission bar, as transmission pulse ($\varepsilon_{t}\left(t\right)$). The whole testing process was recorded in the computer. In addition, the strain gauges shown in Figure 2a were used to measure the change of strain values of the incident and transmission bars, and thus the curves of strain against time were plotted.

**Figure 3:**
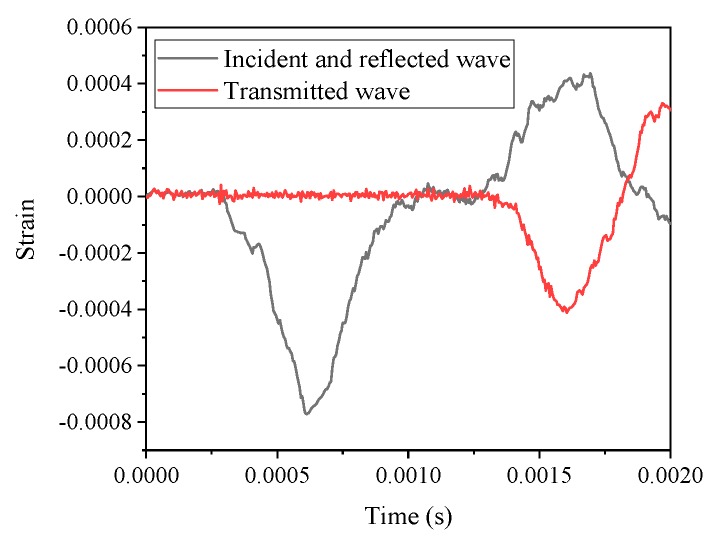
Typical waveforms of incident, reflected and transmitted pulses with a pulse shaper.

The pulse shaping technique was applied to increase the accuracy of the results obtained from SHPB test. The main purpose was to ensure that the tested specimen has sufficient time to reach stress equilibrium by prolonging rising time of incident pulse [7]. A rubber-made slice with a 50 mm in diameter by 2 mm in height was used as a pulse shaper. Figure 3 shows typical waveforms of incident, reflected and transmitted pulses with a pulse shaper.

The stress-strain curve of each mixture can be obtained by inputting the incident pulse ($\varepsilon_{i}\left(t\right)$), reflected pulse ($\varepsilon_{r}\left(t\right)$) and transmission pulse ($\varepsilon_{t}\left(t\right)$). Tri-wave method was used to calculate the stress ($\sigma_{s}\left(t\right)$), strain ($\varepsilon_{s}\left(t\right)$) and strain rate (${\dot{\varepsilon }}_{s}\left(t\right)$) [34,35,36].

#### 2.4.3. SEM Test

The SEM test (Ultra-plus SEM, Jena, Germany) was performed on the fracture pieces after SHPB test to explore the damage morphology of PP fibre. The selected pieces were coated with a gold film before the test. Regarding the image acquisition parameters, the working distance for the SEM test was set in the range of 11.4 to 31.8 mm, while the acceleration voltage was 15 kV. The magnification was in the wide range of 200×.

## 3. Results and Discussion

### 3.1. Workability

**Figure 4:**
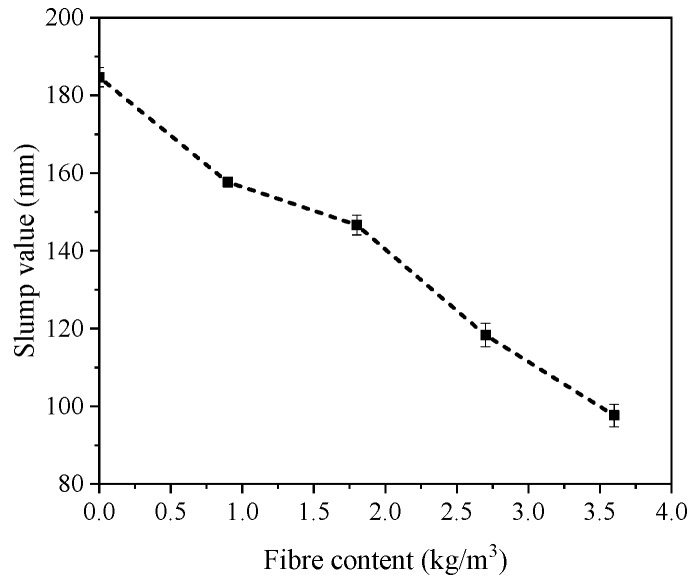
Slump of fibre reinforced concrete with various polypropylene fibre content.

The slump value is normally used to represent the workability of concrete. Figure 4 shows the slump value of all mixtures. It is clear that the slump value of all mixtures was significantly influenced by the PP fibre content. The slump value decreased sharply with increasing PP fibre content in the mixture, which agreed well with the previous studies [3,18,37]. The slump value of PP09, PP18, PP27 and PP36 reduced 14.6%, 20.6%, 36.0% and 47.1%, respectively as compared to the reference mixture (PP00) with a slump value of 184.7 mm. The reduction in workability can be ascribed to the following reasons: (1) adding fibres into the concrete matrix resulted in the increase of viscosity especially when the surface area of fibres are larger [18]; (2) PP fibres exhibit hydrophobic characteristic in nature (i.e., repelling water) and therefore the compactness of mixtures reduced with increasing PP fibre content; (3) clogging of PP fibres would occur, which may affect the uniform dispersion of the fibres in the concrete mixtures [38]; and (4) the internal microstructure of mixtures would be changed due to the addition of PP fibres and thus restrict the flow of mixtures [9]. Since the compactness of concrete may affect its mechanical properties, the dosage of SPs should be adjusted accordingly in order to ensure that all PP fibre reinforced concrete mixtures are workable.

### 3.2. Static Compressive Strength

**Figure 5:**
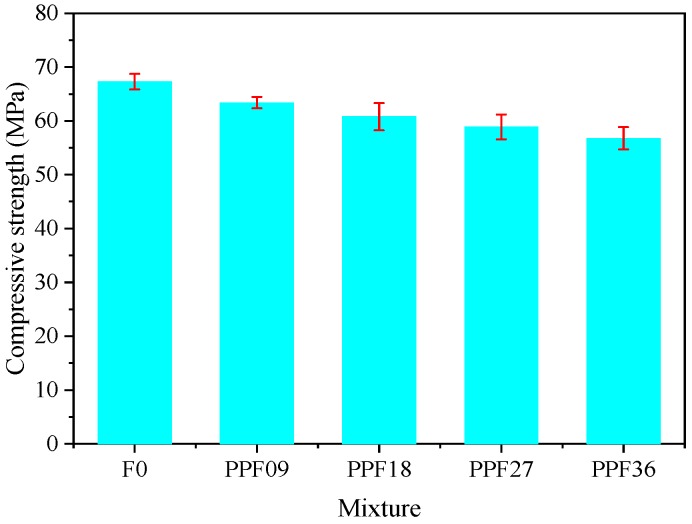
Compressive strength of all mixtures at 28 d.

Figure 5 displays the static compressive strength of all concrete mixtures at testing age of 28 d. It can be seen that the 28 d compressive strengths of PP fibre reinforced concrete mixtures, i.e., PP09 (63.4 MPa), PP18 (60.8 MPa), PP27 (58.9 MPa) and PP36 (56.8 MPa), were lower than that of PP00 (67.3 MPa). This implies that the PP fibre is not capable of enhancing the compressive strength of concrete matrix, which is consistent with previous studies [18,39] pointing out that PP fibres with low stiffness cannot improve the compressive strength of the plain concrete matrix. The reduction of compressive strength became more obvious with the increase of PP fibre dosage. Compared to that of PP00, the compressive strength of PP36 was reduced by 15.6%, which was the highest among all mixtures. In addition, the compressive strengths of PP09, PP18 and PP27 were reduced by 5.8%, 9.7% and 12.5%, respectively as compared to PP00. Therefore, the optimal PP fibre content is determined to be 0.9 kg/m^3^ in terms of static compressive strength. Generally, the reduction in compressive strength after adding PP fibres can be attributed to the hydrophobic characteristic of PP fibre, which affected the dispersion of the fibres in the concrete mixtures and thus the compactness of all PP fibre reinforced concrete was adversely influenced.

### 3.3. Dynamic Compressive Stress-Strain Relationship

#### 3.3.1. Effect of Strain Rate

**Figure 6:**
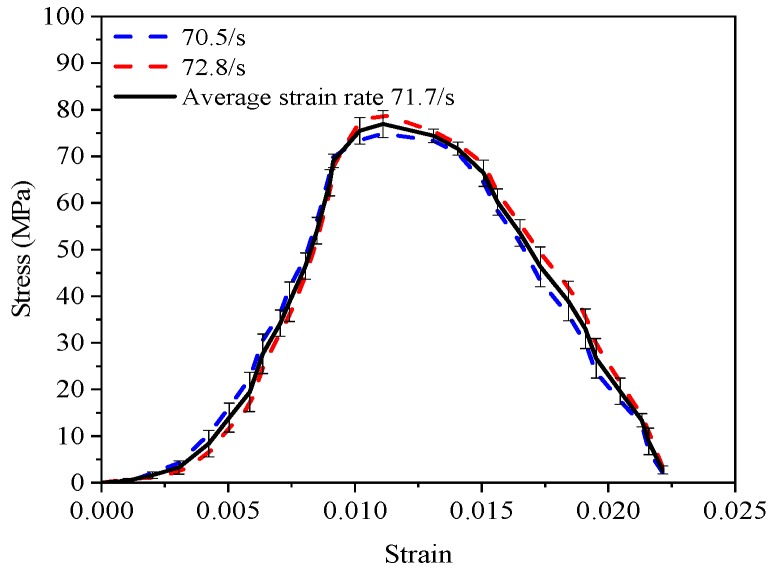
Example of obtaining the dynamic compressive stress-strain curve.

**Figure 7:**
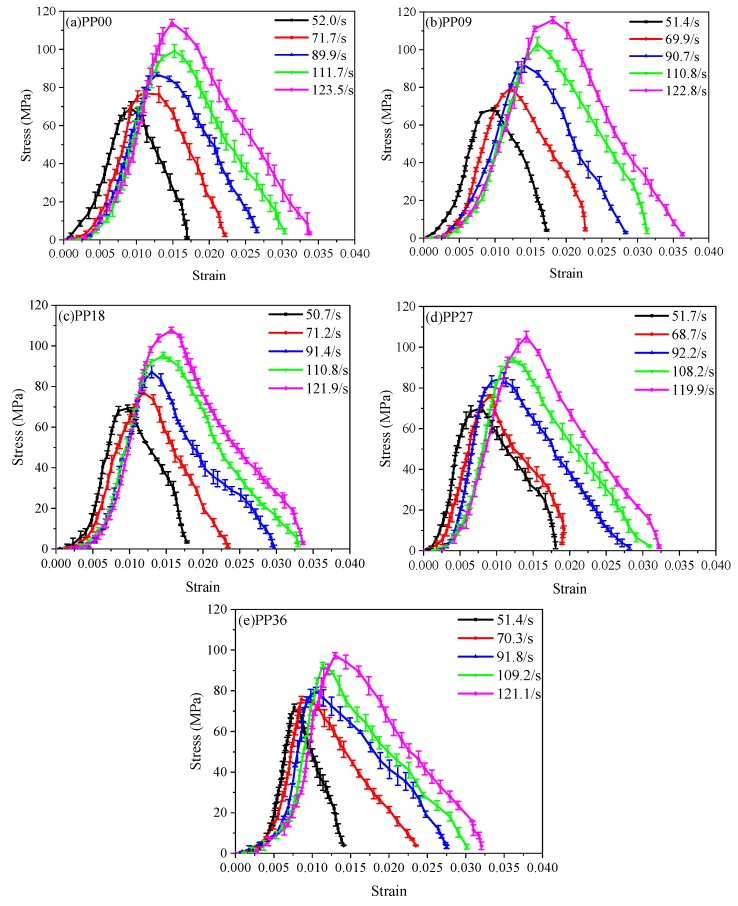
Effect of strain rate on dynamic compressive stress-strain relationship of (**a**) PP00; (**b**) PP09; (**c**) PP18; (**d**) PP27; (**e**) PP36.

Figure 6 shows an example of how the final curves of each mixture presented in Figure 7 was derived. Basically, the final curve was obtained by averaging the curves of two or three specimens, and then the average strain rate was correspondingly determined. The effect of strain rates varying from 50–120 s^−1^ on dynamic compressive stress-strain curves of all mixtures at 28 d are presented in Figure 7. All mixtures are sensitive to strain rate in terms of dynamic compressive stress, known as “strain rate effect”. The dynamic compressive strength increased with the increase of strain rate, which agreed well with previous studies [2,7,9,12,14,17,40]. For instance, peak stress for PP09 was 68.4 MPa with average strain rate of 51.4 s^−1^ and increased to 115.7 MPa with the strain rate 122.8 s^−1^. The increase of dynamic compressive strength due to the “strain rate effect” can be attributed to the lateral inertial effect to the contact surface of the specimens under high-velocity impact loading [9,41,42]. Ross et al. [43] mentioned that sensitivity to strain rate is highly dependent on the moisture condition of concrete. Fu et al. [17] suggested that the “strain rate effect” is mainly ascribed to the combining effect caused by the action of the crack propagation, viscous effect of free water and inertial effect. Therefore, “strain rate effect” can be explained as follows: when the strain rate is lower, the concrete mixtures have sufficient accumulated energy and time for the micro-cracks to propagate into macro-cracks before the final failure of the specimens. The energy required for the propagation of the cracks is lower than that of generating cracks [40], implying that the concrete mixtures need more energy under impact loading, i.e., more cracks appeared under high-velocity impact. However, the time needed for the final failure of concrete mixtures is very short under impact loading. Thus, the accumulated energy of the specimens is lower. The compressive strength is increased only for providing the required energy of generating cracks.

**Figure 8:**
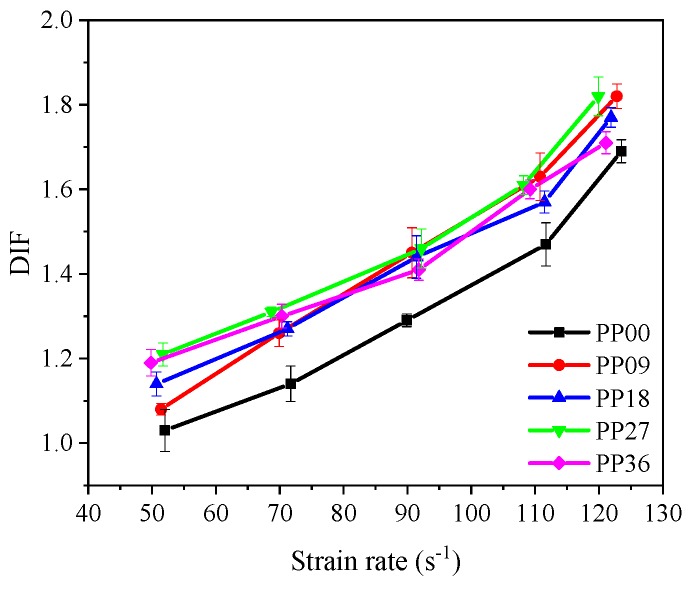
Effect of strain rate on DIF.

Table 7 summarizes the dynamic and static compressive strength, and DIF of all mixtures, where DIF is defined as the ratio of the dynamic compressive strength to the static compressive strength and is widely used to describe the “strain rate effect”. As seen in Figure 8, DIF values of all mixtures increased gradually with the increase of strain rate, showing good agreement with previous studies [9,40]. As the average strain rate increased from 50 s^−1^ to 120 s^−1^, DIF of PP00, PP09, PP18, PP27 and PP36 was increased by 64.1%, 68.5%, 55.3%, 50.4% and 43.7%, respectively. The results suggest that the PP fibre content of 0.9 and 1.8 kg/m^3^ was more efficient in improving dynamic compressive strength of concrete under high-velocity impact loading.

**Figure 9:**
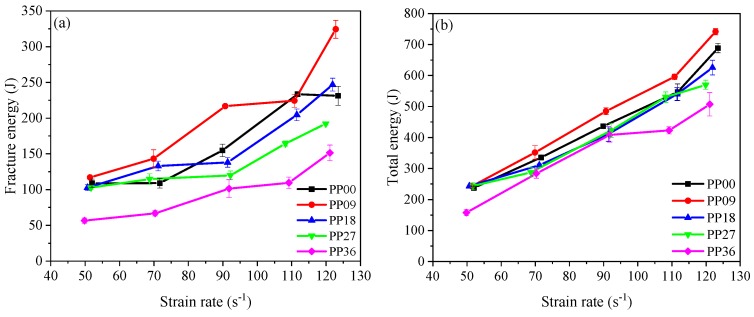
Energy absorption capacity of concrete at different strain rates: (**a**) Fracture energy; (**b**) Tolal energy.

The fracture energy is defined as the energy absorbed by the specimen up to the peak stress. It can be observed from Table 7 that fracture energy was enhanced by the increase of strain rate. Figure 9a shows that PP09 has the maximum fracture energy at the similar strain rate during the impact test. The total energy absorption of all mixtures under impact loading is shown in Figure 9b. For each mixture, the total energy was calculated by multiplying the strain energy density (i.e., the area under the stress-strain curve) by the specimen volume [12]. For each mixture, the energy absorption capacity increased gradually with increasing strain rate, which can also be explained by the “strain rate effect”, that is, the concrete mixtures absorbed more energy under rapid impact loading because of a larger number of cracks. The fracture energy absorption of PP00, PP09, PP18, PP27 and PP36 was enhanced by about 127.8%, 177.0%, 140.2%, 87.1% and 167.5%, respectively when the strain rate increased from around 50 s^−1^ to 120 s^−1^. The total energy absorption of PP00, PP09, PP18, PP27 and PP36 was enhanced by about 190.0%, 205.3%, 156.9%, 133.7% and 221.4%, respectively when the strain rate increased from around 50 s^−1^ to 120 s^−1^.

The deformation capacity of concrete materials can be represented by the strain at peak stress and ultimate strain listed in Table 7. The strain at peak stress and ultimate strain were improved with increasing strain rate. The ultimate strain of each mixture was higher than its strain at peak stress ranging from 83.2% to 173.3%. The ultimate strain of PP09 and PP18 in this study was higher than that of PP00. This can be attributed to the bridging action of the fibres in cracks, which prolongs the strain-softening region and subsequently improves the toughness of FRC.

#### 3.3.2. Effect of Polypropylene Fibre Content

**Figure 10:**
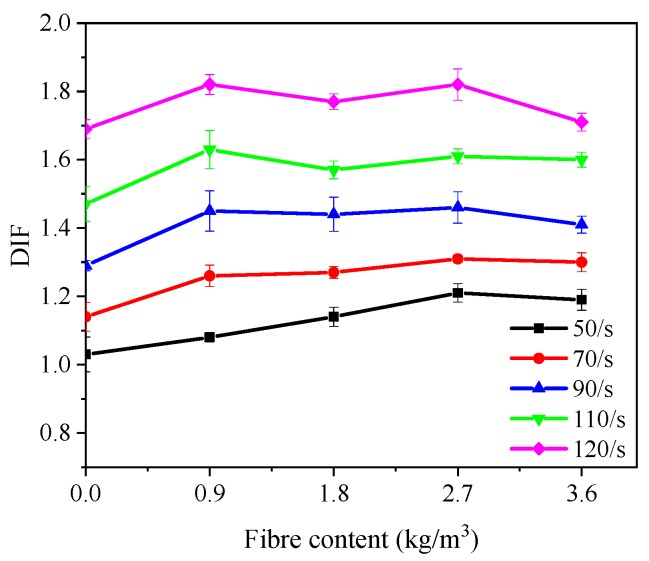
Effect of polypropylene fibre content on DIF.

The effect of PP fibre content on DIF of all concrete mixtures is presented in Figure 10. At the strain rate of around 50 s^−1^, the DIF of PP09, PP18, PP27 and PP36 increased 4.9%, 10.7%, 17.5% and 15.5%, respectively as compared to PP00. When the strain rate was around 70 s^−1^, the DIF of PP09, PP18, PP27 and PP36 enhanced by 10.5%, 11.4%, 14.9% and 14.0% compared to PP00. When the strain rate was around 120 s^−1^, the DIF of PP09, PP18, PP27 and PP36 enhanced by 7.7%, 4.7%, 7.7% and 1.2% compared to PP00. Under the strain rate of 50 s^−1^ and 70 s^−1^, the effect of PP fibre contents on DIF of all mixtures was remarkable. PP09 was found more efficient in controlling the cracking under high strain rate impact loading, which can also be attributed to the short impact loading time under high-velocity impact and thus the contribution of fibres was not remarkable.

**Figure 11:**
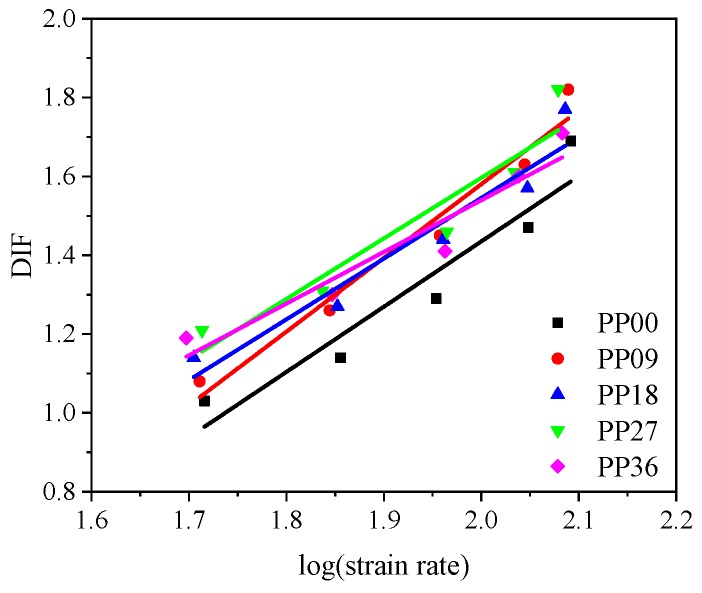
Fitted DIF curves of concrete reinforced with various polypropylene fibre content.

DIF is essential for structural design and getting a reliable DIF is helpful for obtaining an appropriate and economical constructional design [9]. Therefore, it is of importance to obtain a reliable fitted DIF function for each concrete mixture in this study. It was reported by Tedesco et al. [44] that the relationship between DIF and logarithm of strain rate was approximately linear. Following this suggestion, the fitted DIF curves of all concrete mixtures are acquired and demonstrated in Figure 11 as well as Table 8, where the detailed fitted DIF equations for each mixture along with the correlation coefficient (R) are presented. As shown in Table 8, all R^2^ values are greater than 0.9 suggesting that the fitted DIF functions are reliable. The results are consistent with previous study [40]. In addition, it is known that static compressive strength was mainly affected by fibre content, showing reducing tendency with increasing fibre content. On the other hand, for PP09 specimen, although static compressive strength decreased, dynamic compressive strength at relatively low loading rate reduced more remarkably, resulting in a decreased DIF. At high loading rate, DIF increased since with increasing fibre dosage, dynamic compressive strength increased while static compressive strength was reduced.

**Figure 12:**
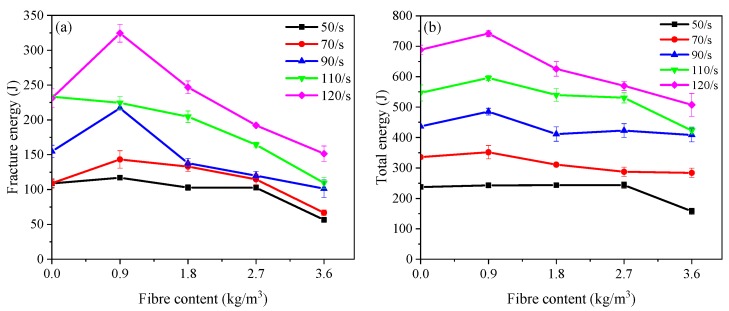
Energy absorption capacity of concrete reinforced with various polypropylene fibre content: (**a**) Fracture energy; (**b**) Total energy.

Figure 12 shows the influence of PP fibre content on energy absorption capacity of concrete. The fracture energy presented is defined as the energy absorbed by the specimen up to the peak stress during the SHPB test, while the total energy reflects the energy absorption capacity in all regions. The fracture energy had an increase up to the PP fibre content of 0.9 kg/m^3^ followed by a gradual decrease with further addition of PP fibre at different strain rates, except 110 s^−1^ strain rate (see Figure 12a). The fracture energy of PP09 was enhanced by 7.7%, 31.4%, 40.1%, −3.0% and 40.3%, respectively at the average strain rate around of 50 s^−1^, 70 s^−1^, 90 s^−1^, 110 s^−1^ and 120 s^−1^ compared to that of the reference mixture (PP00). Figure 12b shows the development of total energy of PP fibre mixtures with varying content. A similar tendency can be seen that the PP fibre content of 0.9 kg/m^3^ corresponds to the maximum total energy absorption capacity and thus it can be regarded as the optimal dosage regarding the energy absorption capacity.

The mechanical properties of FRC are highly dependent on its internal structure and the properties of fibres and cement matrix. The number of fibres inside the matrix can affect the spatial spacing of the fibres. It was reported that the static mechanical properties were influenced by space between fibres inside the matrix. For instance, the tensile strength decreased with increasing fibre spacing [45]. Based on the current experimental results, it is found that the static mechanical properties showed an exactly dissimilar trend with that of dynamic properties especially with relatively high strain rate. When the strain rate increases, the loading time is very short. Under such conditions, the FRC specimen may only exhibit the optimal dynamic compressive properties when its internal structure reaches the optimal state. In this work, the optimal fibre content in terms of workability, static and dynamic compressive properties was found to be 0.9 kg/m^3^ (i.e., PP09), as PP09 presented the best workability among all mixtures and its static compressive strength was not affected significantly (i.e., only 5.8% reduction as compared to PP00). In addition, the DIF of PP09 increased by 7.7% at the strain rate of 120 s^−1^ as compared to PP00, which is the highest among all mixtures.

### 3.4. Dynamic Failure Mode

**Figure 13:**
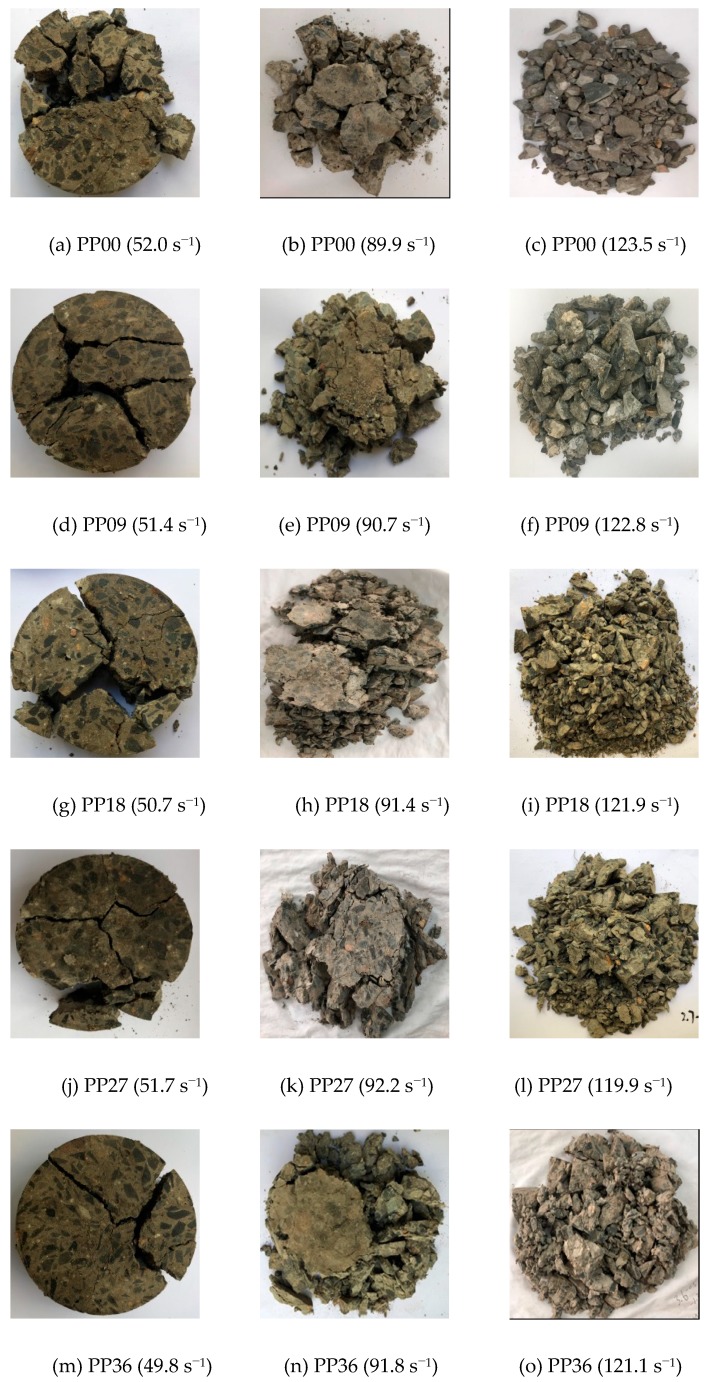
Effects of strain rate and polypropylene fibre dosage on failure mode of all mixtures.

The effect of strain rate on the dynamic failure mode of all mixtures is illustrated in Figure 13. It is found that the failure pattern of all mixtures changed from splitting failure into pulverization failure with increasing strain rate, which is consistent with previous studies [2,7,9,12]. As shown in Figure 13a,d,g,j,m, several big broken fragments were observed for all mixtures when the strain rate is lower. However, when the strain rate is relatively high, all deformed specimens are broken into many tiny fragments (see Figure 13c,f,i,l,o). This phenomenon is in consistence with the “strain rate effect”, which can also be attributed to the extremely short loading time under high-velocity impact loading.

**Figure 14:**
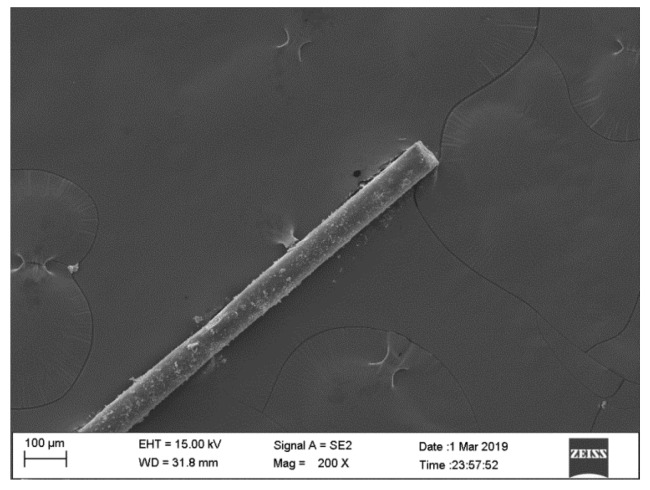
SEM micrographs showing the original morphology of PPF.

**Figure 15:**
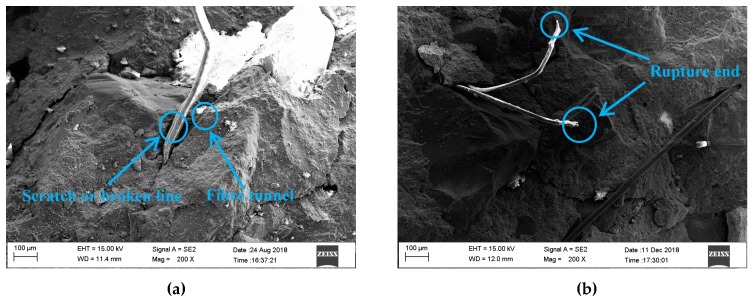
SEM micrographs showing the morphology of PPF after impact: (**a**) Scratch or broken line and fibre tunnel; (**b**) Rupture end.

It can be clearly observed that the failure patterns of the mixtures were more complete with the PP fibre under low strain rate. As shown in Figure 13d,g,j,m, only several longitudinal splitting failures were observed. However, PP00 exhibited a brittle failure pattern. The results suggested that the PP fibre can restrict the growth of cracks under low strain rate. When the strain rate is relatively high (e.g., 120 s^−1^), PP fibres were not so efficient to control cracking of mixtures as pulverization failure pattern was observed for all mixtures. It generally requires two parts of energy to break a fibre reinforced concrete, i.e., the energy to break the matrix and the energy to pull out or rupture fibre reinforcements [9]. The SEM images presented in Figure 14 and Figure 15 can be used to compare the original morphology of PP fibres with the failure morphology in the specimen after impact test. As seen in Figure 15a, PP fibres were found to be pulled out of the concrete matrix, resulting in scratches and damage on the surface of the fibres. Figure 15b shows that PP fibres were ruptured in the process of specimen fracture. When the strain rate is relatively low, the generated micro-cracks had enough time to reach the transition zone between cement matrix and aggregate resulting in complete failure of the specimen. Thus, PP00 (without fibre reinforcement) failed quickly as compared to other PP fibre reinforced concrete mixtures. With the incorporation of PP fibres, many micro-cracks were restricted by the fibres and thus the ductility of the specimen enhanced. As a result, the failed specimens exhibited more ductile failure pattern compared to that of PP00. When the strain rate is relatively high, the impact loading time is extremely short and therefore the dynamic compressive strength of all mixtures was enhanced by increasing the energy to generate cracks. Although PP fibres can help to resist some cracks, the efficiency would be lower due to the extremely high impact velocity.

## 4. Conclusions

In this study, the workability, static compressive strength and dynamic compressive properties of concrete reinforced with PP fibres with various fibre content (i.e., 0.9, 1.8, 2.7 and 3.6 kg/m^3^) were investigated and compared with those of the reference mixture without fibre (PP00). Compared with the previous researches concerning the dynamic properties of PP fibre reinforced normal strength concrete, the dynamic properties of high strength concrete with series content of PP fibres was emphasized here. Based on the experimental results, the main conclusions are drawn as follows:

- The incorporation of PP fibres enhanced the dynamic compressive behaviour but reduced the workability and static compressive strength of concrete mixtures. PP36 exhibited the lowest workability among all mixtures, where its workability reduced 14.6% as compared to PP00. The optimal PP fibre content was found 0.9 kg/m^3^ (i.e., PP09) in terms of workability, and static and dynamic compressive properties as PP09 exhibited the best workability among all PP fibre reinforced concrete mixtures and showed the least reduction (5.8%) in static compressive strength and an increase of 7.7% in DIF when the strain rate was120 s^−1^ compared to PP00.
- The ‘strain rate effect’ can be observed for all mixtures regarding the dynamic compressive properties. As strain rate increased, dynamic compressive strength of all mixtures increased and the DIF of all mixtures was enhanced gradually.
- High correlation coefficients of the fitted DIF curves can be found for all mixtures, which indicates that the proposed equations can be used to describe well the relationship between the DIF and strain rate of PP fibre reinforced concrete under impact loading.
- When the strain rate is relatively low, PP fibres were efficient in controlling the growth of micro-cracks under impact loading. However, the effectiveness of PP fibres was reduced when the strain rate was increased, which can be mainly attributed to the extremely short impact loading time.

## Figures and Tables

**Table 1:** Chemical compositions of cement.

Oxide	SiO_2_	Al_2_O_3_	CaO	Fe_2_O_3_	SO_3_	MgO	Na_2_O	K_2_O	LOI
Cement	21.35	5.98	60.56	2.91	2.05	2.22	0.21	0.75	3.97

**Table 2:** The gradation compositions of the fine aggregate.

**Sieve Size (mm)**	4.75	2.36	1.18	0.60	0.30	0.15	0.075
**Total Percentage Retained (%)**	0	10.5	27.8	51.5	76.2	90.8	98.6

**Table 3:** The gradation compositions of the coarse aggregate.

**Sieve Size (mm)**	20.0	19.0	16.0	13.2	9.5	4.75
**Total Percentage Retained (%)**	0	2.6	18.3	55.3	85.4	98.1

**Table 4:** Properties of polypropylene fibre.

Fibre Type	Length (mm)	Diameter (μm)	Fibre Strength (MPa)	Density (kg/m^3^)	Modulus of Elasticity (GPa)
PP	19	26	376	910	3.79

**Table 5:** Mixture proportions.

No.	Symbol	Cement (kg/m^3^)	Water (kg/m^3^)	FA (kg/m^3^)	CA (kg/m^3^)	SPs (kg/m^3^)	PP Fibre (kg/m^3^)	*V_f_* (%)
1	PP00	569	136	645	1100	5.69	0.0	/
2	PP09	569	136	645	1100	5.69	0.9	0.1
3	PP18	569	136	645	1100	5.69	1.8	0.2
4	PP27	569	136	645	1100	5.69	2.7	0.3
5	PP36	569	136	645	1100	5.69	3.6	0.4

Note: FA (Fine Aggregate); CA (Coarse Aggregate); SPs (Superplasticisers); PP (Polypropylene).

**Table 6:** Number of static specimens, dynamic specimens and strain rate.

Symbol	Number of Specimens		Strain Rate Rype (s^−1^)				
	Static Specimens	Dynamic Specimens	50	70	90	110	120
PP00	3	15	3	3	3	3	3
PP09	3	15	3	3	3	3	3
PP18	3	15	3	3	3	3	3
PP27	3	15	3	3	3	3	3
PP36	3	15	3	3	3	3	3

**Table 7:** Summary of dynamic and static properties of PP fibre reinforced concrete.

Symbol	Average Strain Rate (s^−1^)	Maximum Dynamic Stress (MPa)	Strain at Peak Stress (×10^−3^)	Ultimate Strain (×10^−3^)	Static Compressive Strength (MPa)	DIF	Fracture Energy (J)	Total Energy (J)
PP0	52.0	69.1 ± 3.45	9.25	16.95	67.3 ± 1.46	1.03 ± 0.051	108.7 ± 5.92	237.4 ± 3.81
	71.7	76.9 ± 2.86	11.11	22.14	67.3 ± 1.46	1.14 ± 0.042	109.0 ± 6.97	335.6 ± 6.50
	89.9	86.8 ± 1.00	12.82	26.54	67.3 ± 1.46	1.29 ± 0.015	154.8 ± 8.67	436.5 ± 1.97
	111.7	99.1 ± 3.46	15.22	30.35	67.3 ± 1.46	1.47 ± 0.051	233.4 ± 2.05	546.6 ± 26.44
	123.5	113.9 ± 1.80	14.88	33.78	67.3 ± 1.46	1.69 ± 0.027	231.3 ± 13.33	688.5 ± 15.02
PP09	51.4	68.4 ± 0.86	9.78	17.25	63.4 ± 1.06	1.08 ± 0.014	117.1 ± 0.39	242.9 ± 3.97
	69.9	79.8 ± 1.97	12.19	22.68	63.4 ± 1.06	1.26 ± 0.031	143.2 ± 12.53	351.6 ± 21.94
	90.7	91.7 ± 3.74	14.13	28.33	63.4 ± 1.06	1.45 ± 0.059	216.8 ± 0.18	485.1 ± 11.15
	110.8	103.1 ± 3.54	15.95	31.31	63.4 ± 1.06	1.63 ± 0.056	224.4 ± 9.08	595.9 ± 8.41
	122.8	115.7 ± 1.84	18.09	36.29	63.4 ± 1.06	1.82 ± 0.029	324.4 ± 12.66	741.5 ± 10.19
PP18	50.7	69.2 ± 1.69	9.76	17.83	60.8 ± 2.52	1.14 ± 0.028	102.8 ± 4.16	243.4 ± 0.77
	71.2	77.1 ± 1.05	11.89	23.38	60.8 ± 2.52	1.27 ± 0.017	133.0 ± 6.83	310.7 ± 3.68
	91.4	87.3 ± 3.05	13.04	29.69	60.8 ± 2.52	1.44 ± 0.050	138.0 ± 6.66	411.3 ± 23.52
	111.5	95.3 ± 1.60	14.63	32.95	60.8 ± 2.52	1.57 ± 0.026	204.6 ± 8.19	540.0 ± 20.91
	121.9	107.7 ± 1.41	15.71	36.62	60.8 ± 2.52	1.77 ± 0.023	246.9 ± 8.97	625.4 ± 23.76
PP27	51.7	70.1 ± 1.55	7.99	18.06	57.9 ± 2.31	1.21 ± 0.027	102.7 ± 2.68	243.8 ± 10.60
	68.7	76.0 ± 0.64	9.08	23.44	57.9 ± 2.31	1.31 ± 0.011	114.7 ± 7.33	287.4 ± 15.03
	92.2	84.8 ± 2.64	10.98	28.14	57.9 ± 2.31	1.46 ± 0.046	119.9 ± 6.08	422.8 ± 22.37
	108.2	93.7 ± 1.27	12.24	30.88	57.9 ± 2.31	1.61 ± 0.022	164.4 ± 3.44	530.3 ± 16.80
	119.9	105.2 ± 2.66	14.07	32.22	57.9 ± 2.31	1.82 ± 0.046	192.2 ± 1.80	569.8 ± 15.11
PP36	49.8	67.6 ± 1.78	7.67	14.09	56.8 ± 2.06	1.19 ± 0.031	56.6 ± 3.70	157.8 ± 10.10
	70.3	73.6 ± 1.58	8.60	23.50	56.8 ± 2.06	1.30 ± 0.028	66.8 ± 3.93	283.9 ± 15.14
	91.8	80.2 ± 1.40	10.48	27.50	56.8 ± 2.06	1.41 ± 0.025	101.3 ± 12.50	408.3 ± 22.85
	109.2	90.7 ± 1.27	11.35	30.12	56.8 ± 2.06	1.60 ± 0.022	109.5 ± 7.99	423.0 ± 11.74
	121.1	97.3 ± 1.48	12.99	32.04	56.8 ± 2.06	1.71 ± 0.026	151.4 ± 10.98	507.2 ± 37.78

**Table 8:** Summary of the fitted DIF equations for different mixtures.

No.	Symbol	Fitted Equation of DIF	R^2^
1	PP00	$\mathrm{DIF}=1.656\log\dot{\varepsilon }-1.877$	0.911
2	PP09	$\mathrm{DIF}=1.871\log\dot{\varepsilon }-2.161$	0.966
3	PP18	$\mathrm{DIF}=1.541\log\dot{\varepsilon }-1.536$	0.928
4	PP27	$\mathrm{DIF}=1.537\log\dot{\varepsilon }-1.477$	0.902
5	PP36	$\mathrm{DIF}=1.312\log\dot{\varepsilon }-1.084$	0.922
